# Supplementary material for: Oncolytic Adenovirus Armed with a Novel Agonist of the CD137 Immune Checkpoint Stimulator Suppresses Tumor Growth
Source: Vaccines (Basel). 2024 Mar 21;12(3):340. doi: 10.3390/vaccines12030340 (PMC10974162; doi:10.3390/vaccines12030340)
Supplement: Supplementary file 1 [file vaccines-12-00340-s001.zip › vaccines-2857742-supplementary.docx]

**Supplementary Material**

| No. | ANTIBODY | COMPANY | CATALOG |
| --- | --- | --- | --- |
| 1 | LIVE/DEAD™ Fixable Near-IR Dead Cell Stain Kit | Invitrogen | L34975 |
| 2 | V500 Syrian Hamster anti mouse CD3e | BD Horizon | 560771 |
| 3 | PE/Dazzle™ 594 anti-mouse CD4 Antibody | BioLegend | 100455 |
| 4 | FITC anti-mouse CD8a Recombinant Antibody | BioLegend | 155004 |
| 5 | Brilliant Violet 650™ anti-mouse/human CD11b Antibody | BioLegend | 101239 |
| 6 | AF647 Anti-mouse CD11c | BioLegend | 117312 |
| 7 | BV570 Anti-mouse CD19 | BioLegend | 115535 |
| 8 | BB700 Anti-mouse CD44 | BD Horizon | 566506 |
| 9 | APC Fire810 Anti-mouse CD45 | BioLegend | 103174 |
| 10 | BV711 Anti-mouse CD62L | BioLegend | 104445 |
| 11 | PE/Cy7 Anti-mouse CD107a | BD Pharmingen | 560647 |
| 12 | PE anti-mouse CD137 Antibody | BioLegend | 106105 |
| 13 | AF700 Anti-mouse NK1.1 | BioLegend | 108730 |
| 14 | Pacific Blue Anti-mouse F4/80 | BioLegend | 123124 |
| 15 | PE/Cy5 Anti-mouse CD25 | BioLegend | 102010 |

**Supplementary Table 1. List of antibodies and live dead kit used as an immune cell phenotyping panel for flow cytometry.** In the table above we describe the antibodies used for each immune cellular marker analyzed by flow cytometry. This panel was generated using the Cytek Cloud Panel Builder to adjust individual wavelengths and intensities for each dye used.


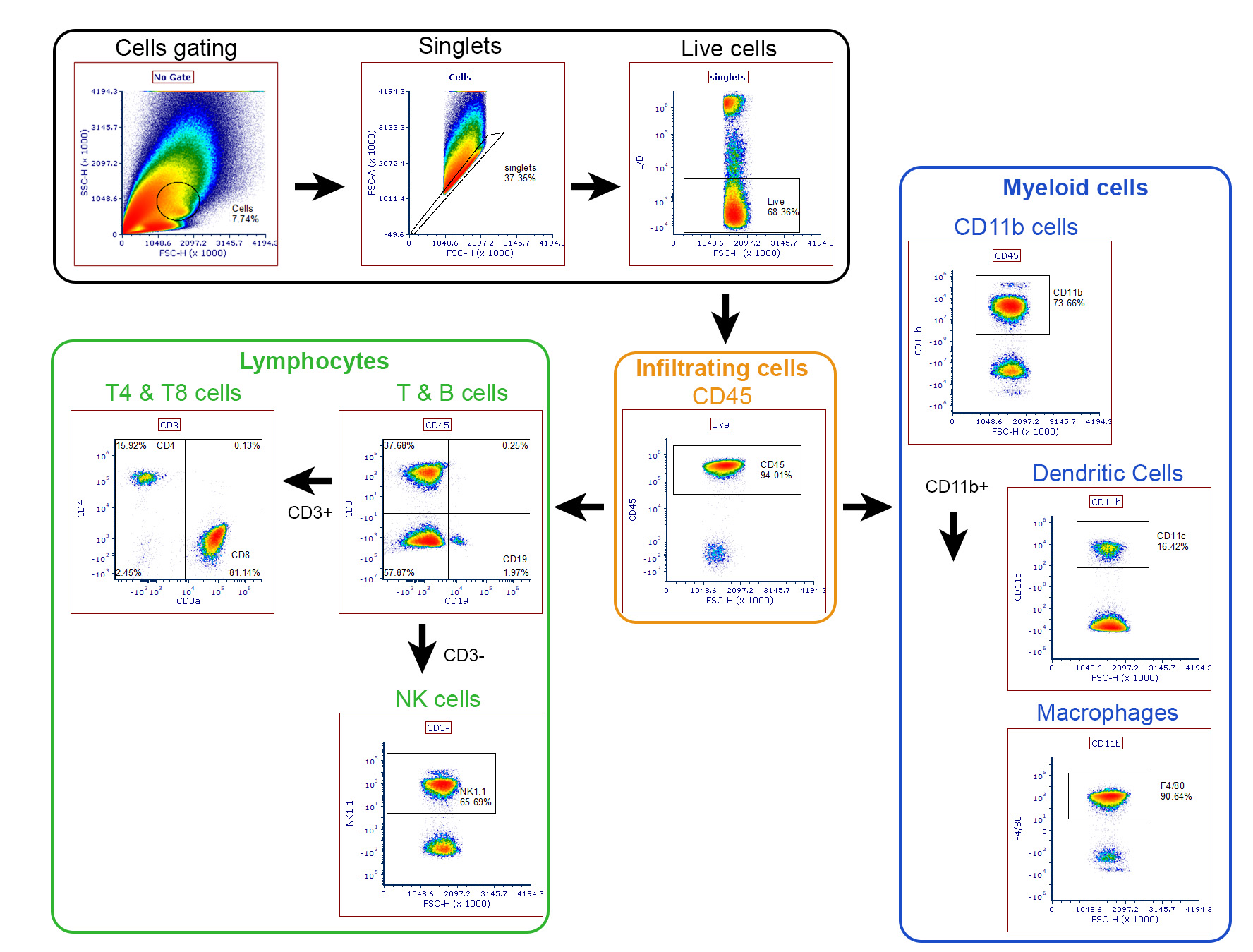


**Supplementary Figure 1. Flow cytometry gating strategy used to identify the immune cell populations.** From the single cell suspension obtained from the tumor samples we performed a flow cytometry analysis to identify the different tumor infiltrating immune cells populations as described in this gating strategy.


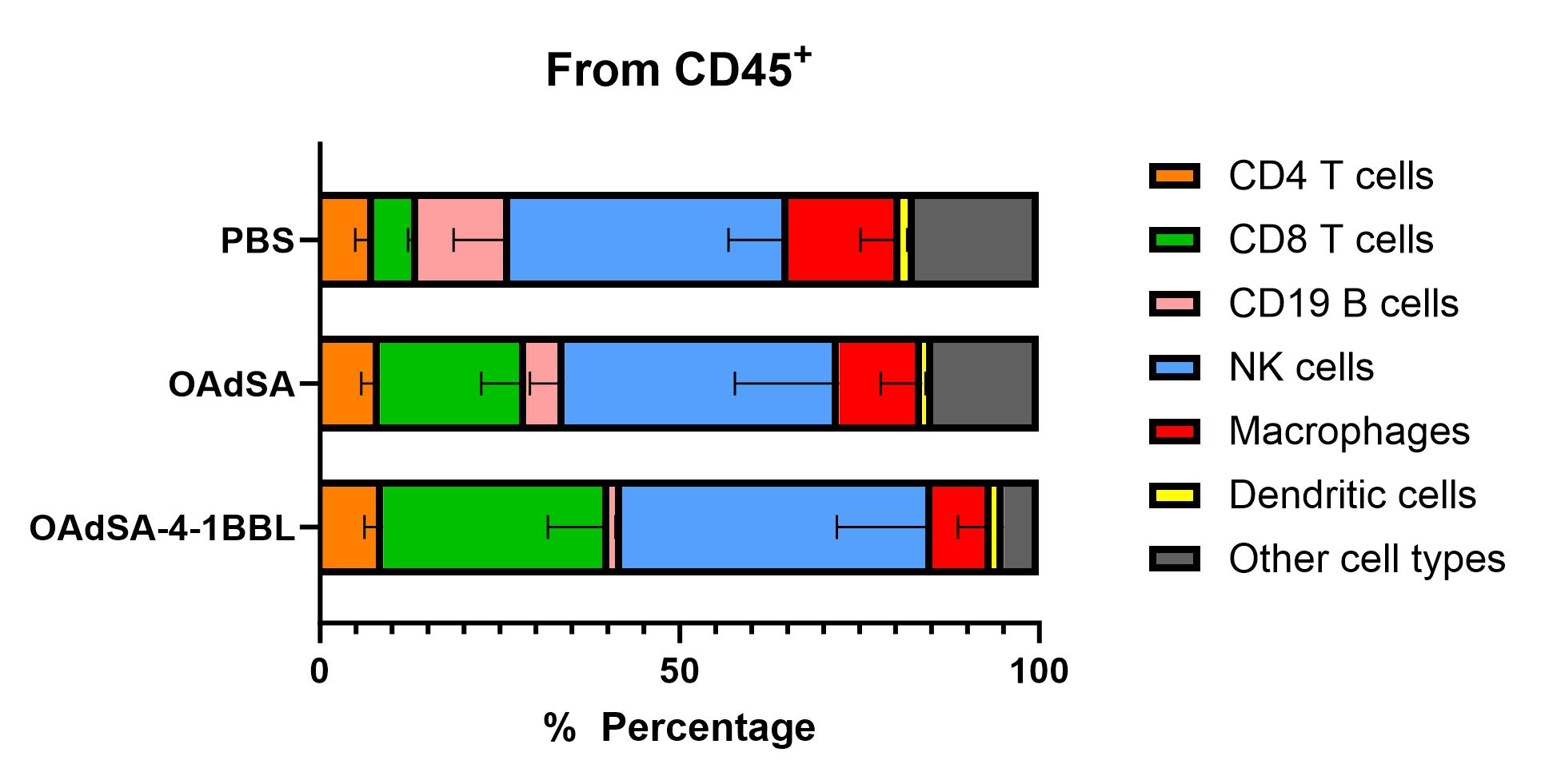


**Supplementary Figure 2. Immune cell populations percentage from the total infiltrating cells in tumors (CD45^+^ gating).** Comparison between the immune cell populations percentage/proportions obtained from the total tumor infiltrating cells analyzed by flow cytometry (gating from CD45^+^).


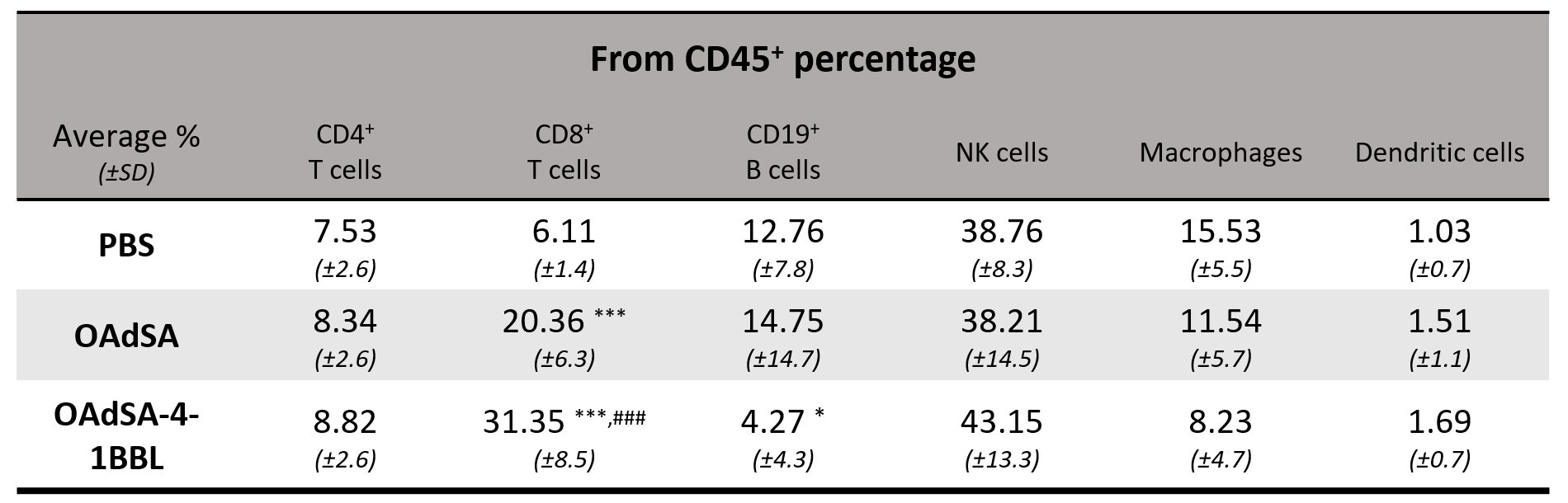


**Supplementary Table 2. The percentage of total infiltrating cells from tumors for each immune cell population.** Table shows the average percentage obtained for each infiltrating immune cell’s population, (±SD). * vs PBS p<0.05, *** p<0.001, ### vs OAdSA p<0.001.
